# Strategies and Best Practices to Improve Diversity, Equity, and Inclusion Among US Graduate Medical Education Programs

**DOI:** 10.1001/jamanetworkopen.2022.55110

**Published:** 2023-02-08

**Authors:** Dowin Boatright, Maya London, Arra Jane Soriano, Marjorie Westervelt, Stephany Sanchez, Jed D. Gonzalo, William McDade, Tonya L. Fancher

**Affiliations:** 1 Department of Emergency Medicine, Yale University, New Haven, Connecticut; 2 now with Department of Emergency Medicine, New York University Grossman School of Medicine, New York, New York; 3 currently a medical student at University of California, Davis, School of Medicine; 4 Center for a Diverse Healthcare Workforce, University of California, Davis, School of Medicine; 5 Office of Medical Education, University of California, Davis, School of Medicine; 6 Department of Internal Medicine, University of California, Davis, School of Medicine; 7 Department of Internal Medicine, Virginia Tech Carilion School of Medicine, Roanoke; 8 Accreditation Council for Graduate Medical Education, Chicago, Illinois

## Abstract

**Importance:**

Closing the diversity gap is critical to ensure equity in medical education and health care quality. Nevertheless, evidence-based strategies and best practices to improve diversity, equity, and inclusion (DEI) in the biomedical workforce remain poorly understood and underused. To improve the culture of DEI in graduate medical education (GME), in 2020 the Accreditation Council of Graduate Medical Education (ACGME) launched the Barbara Ross-Lee, DO, Diversity, Equity, and Inclusion Award to recognize exceptional DEI efforts in US residency programs.

**Objective:**

To identify strategies and best practices that exemplary US GME programs use to improve DEI.

**Design and Setting:**

This qualitative study performed an exploratory content analysis of award applications submitted to the ACGME over 2 cycles in 2020 and 2021, using the constant comparative method. The research team first acknowledged their own biases related to DEI, used caution to not overinterpret the data, and performed several cross-checks during data analysis to ensure confirmability of the results. A preliminary codebook was developed and used during regular adjudication sessions. Disagreements were discussed until agreements were reached.

**Main Outcomes and Measures:**

Foundational (ie, commonly cited, high-impact, and small-effort strategies considered achievable by all programs) and aspirational (ie, potential for high impact but requiring greater effort and investment) DEI strategies used by exemplary GME programs.

**Results:**

This qualitative study included 29 award applications submitted between August 17, 2020, and January 11, 2022. Strategies spanned the education continuum from premedical students through faculty. Foundational strategies included working with schools, community colleges, and 4-year college campuses; providing structured support for visiting students; mission-driven holistic review for admissions and selection; interviewer trainings on implicit bias mitigation and on how racism and discrimination impact admission processes and advancement; interview-day DEI strategies; inclusive selection and DEI committees; mission statements that include DEI; and retention efforts to improve faculty diversity. Aspirational strategies included development of longitudinal bidirectional collaborations (eg, articulation agreements, annual workshops, funded rotations and/or research) with organizations working with applicants who were historically excluded and underrepresented in medicine, blinding metrics in residency applications, longitudinal curricula on DEI and health equity, and faculty mentoring such as affinity groups, mentored research, and joint academic-community recruitments. Findings provide residency program leadership with a menu of options at various inflection points to foster DEI within their programs.

**Conclusions and Relevance:**

The findings of this qualitative study suggest that GME programs might adopt strategies of exemplary programs to improve DEI in residency, ensure compliance with accreditation standards, and improve health outcomes for all.

## Key Points

**Question:**

What strategies and best practices do US graduate medical education (GME) programs use to improve diversity, equity, and inclusion (DEI) in residency?

**Findings:**

In this qualitative study of the Accreditation Council of Graduate Medical Education’s Barbara Ross-Lee, DO, Diversity, Equity, and Inclusion Award submissions, foundational and aspirational DEI strategies used by exemplar GME programs were identified. Foundational strategies included mission-driven holistic review for admissions and selection, interviewer trainings on implicit bias mitigation and on how racism and discrimination affect admission processes and advancement, inclusive selection and DEI committees, mission statements that include DEI, and retention efforts to improve faculty diversity.

**Meaning:**

These findings suggest that GME programs might adopt strategies of exemplar programs to improve DEI in residency, ensure compliance with accreditation standards, and improve health outcomes for all.

## Teaser

This qualitative study performs an exploratory content analysis to investigate what strategies and best practices are used by graduate medical education programs to improve diversity, equity, and inclusion in their programs with detailed narratives from residency program applicants.

## Introduction

Closing the health care workforce diversity gap is critical to ensure equity in access to medical education and to improve health care quality.^1,2,3,4^ Nevertheless, evidence-based strategies and best practices to improve diversity, equity, and inclusion (DEI) in the biomedical workforce remain poorly understood and underused.

To improve the culture of DEI in graduate medical education (GME) in the US, the Accreditation Council of Graduate Medical Education (ACGME) introduced a diversity accreditation standard starting in 2019. This policy standard mandates that all GME training programs engage in “practices that focus on mission-driven, ongoing, systematic recruitment and retention of a diverse and inclusive workforce*.*”^5^ Little is known about how programs are addressing the standard.

To recognize efforts to achieve DEI, the ACGME launched the Barbara Ross-Lee, DO, Diversity, Equity, and Inclusion Award.^6^ Using the detailed narratives from ACGME-accredited residency programs applying for the Barbara Ross-Lee, DO, Diversity, Equity, and inclusion award, we performed an exploratory content analysis to investigate the following research question: “What strategies and best practices do GME programs use to improve DEI in their programs?”

## Methods

### Study Setting and Data Source

This qualitative study followed the Standards for Reporting Qualitative Research (SRQR) reporting guideline and was deemed exempt from further review by the Institutional Review Board at the Yale University School of Medicine because it was not considered human participant research. Each winter starting in 2020, the ACGME issues a call for applications for the Barbara Ross-Lee, DO, Diversity, Equity, and Inclusion Award.^6^ US residency programs, sponsoring institutions, and specialty organizations are eligible to apply. Applicants describe program efforts to increase diversity in GME and pre-GME (inclusive of any level of learner before they enter GME) and assessment of their impact (including letters from trainees, faculty, and institutional leadership). In consultation with the ACGME’s Department of DEI, a research task force led by two of us (D.B. and T.L.F.) analyzed all applications submitted by GME programs (only) in 2020 (n = 22) and 2021 (n = 7) to develop a roadmap of strategies and best practices. Applications ranged from 9 to 152 pages in length. The research team was unaware of which applicants received awards and reviewed all applications as self-nominated exemplars.

Programs used the terms *underrepresented minority* (URM) and *underrepresented in medicine* (URiM) to describe communities involved in their efforts. We retained terms used by each program and preferentially used the phrases *historically excluded* and *UriM* when making overarching comments about the study findings.

### Study Approach

Since limited literature exists regarding a cohesive framework of strategies for GME programs, we used an inductive approach. Although we were open to exploratory and yet-to-be identified results, we were sensitized in our approach to the Kellogg Logic Model framework, which allows for the systematic identification of activities, outputs, outcomes, and impact of any program development or implementation^7^; our analytic approach included these 4 categories in our analysis. Three members of the research team with prior experience in qualitative research (D.B., M.W., and J.D.G.) led the analytical approach. Members of the team had expertise in ACGME (W.M.), GME (S.S.), workforce diversity (T.L.F.), and pre-GME DEI (M.L. and A.J.S.).

### Data Analysis

At the beginning of our analysis, our research team acknowledged our own biases related to DEI, inclusive of our preconceived notions about the importance and benefit of pursuing improvements in DEI, strategies that may work best, and/or the limitations of pursuing DEI initiatives based on our own experience in local environments.^8^ Therefore, we used caution to not overinterpret the data, and we sought to ask neutral questions during research team adjudication sessions. We additionally performed several cross-checks during data analysis to ensure confirmability of our results.

We approached the data using the constant comparative method.^9^ First, to develop a preliminary codebook, all investigators independently reviewed 2 award submissions (total 22 pages) to identify general categories. In 2 subsequent team meetings, a preliminary codebook of categories was identified and subsequently used in the analysis. Two investigators (M.L. and M.W.) then analyzed a small sample of the applications (10 applications, total of 60 pages), further identifying examples within each category and initial themes and modifying the codebook. The research team held regular adjudication sessions to discuss the categories and themes. Pairs of investigators then independently analyzed all data, with continued adjudication sessions (total of 8 meetings and 12 hours). In these meetings, initial categories and themes were compared for consistency and agreement, and disagreements were discussed until agreements were reached. The discussions also allowed for the deliberation over disagreements, collapsing and modification of codes and categories, and the creation of a conceptual model related to the time phases of DEI strategies, all of which enhanced the credibility of this work.^8^ Consensus for all disagreements and findings was reached through group discussion. All investigators agreed on final categories, themes, and conceptual models. A web application for managing, analyzing, and presenting qualitative research data (Dedoose, version 8.0.35) was used for data management.^10^ The investigators categorized strategies as foundational or aspirational. Strategies were considered foundational if they were commonly cited and had the potential for high impact with small effort, and thereby achievable by all GME programs. The remaining strategies were considered aspirational, with the potential for high impact but requiring greater effort and investment.

## Results

Between August 17, 2021, and January 11, 2022, a total of 29 applications were available for analysis, including 4 applications in 2022 submitted by residency programs that also applied in 2021 (25 programs). Of these applications, 17 were from primary care GME programs (internal medicine, pediatrics, or family medicine), 3 were from surgical GME programs (general surgery or obstetrics and gynecology), and 5 were from other GME programs (emergency medicine, psychiatry, or anesthesiology) (Table 1). Programs were geographically distributed across the US, ranged in size from less than 30 to 90 or more residents, and were affiliated with public and private medical schools or had no medical school affiliation.^11^ Of the 23 programs affiliated with a medical school, 10 were ranked among the top 20 schools with National Institutes of Health funding by the Blue Ridge Institute for Medical Research.^12^ Programs described DEI efforts focused on residents who were racially or ethnically underrepresented (URM or URiM) and less commonly described efforts focused on LGBTQAI (lesbian, gay, bisexual, transgender, queer [or questioning], asexual [or allied], intersex) communities.^13,14,15,16^ There were no thematic differences in the approach to DEI across specialties. Our analysis identified a phase-based sequence of DEI efforts across the educational continuum. Below we highlight common themes and practices among 15 foundational and 18 aspirational strategies and offer suggestions that all GME programs might consider to promote DEI.

**Table 1.** Characteristics of Applications and Graduate Medical Education Program Applicants to the Accreditation Council of Graduate Medical Education’s Barbara Ross-Lee, DO, Diversity, Equity, and Inclusion Awards in 2020 and 2021

Characteristic	Total No. (%)
Applications per cycle^a^	
2020	22 (75.9)
2021	7 (24.1)
Program characteristics	
Program specialty^b^	
Primary care	17 (68.0)
Nonprimary care	8 (32.0)
Geographic region^c^	
Pacific West	9 (36.0)
Mountain West	1 (4.0)
West North Central	1 (4.0)
East North Central	2 (8.0)
West South Central	1 (4.0)
East South Central	0
South Atlantic	3 (12.0)
Middle Atlantic	6 (24.0)
New England	2 (8.0)
Program size (total No. of residents)^c^	
≤30	7 (28.0)
31-90	8 (32.0)
>90	10 (40.0)
Medical school affiliation^c^	
Public	9 (36.0)
Private	14 (56.0)
None or independent	2 (8.0)
Medical school affiliate NIH research funding^d^	
Top 20	10 (43.5)
Not top 20	13 (56.5)

Abbreviation: NIH, National Institutes of Health.

^a^ In 2021, 4 programs from 2020 were reapplicants. Programs are counted only once in program characteristics section (n = 25).

^b^ Primary care included family medicine, pediatrics, and internal medicine. Nonprimary care included all other specialties in the applicant pool: anesthesiology, emergency medicine, obstetrics and gynecology, and surgery.

^c^ Determined using the American Medical Association FRIEDA database.^11^

^d^ Determined by the 2021 Blue Ridge Institute for Medical Research rankings.^12^

### Themes, Strategies, and Practices to Develop an Organizational Culture That Emphasizes DEI

Our analysis identified numerous foundational and aspirational strategies used by GME programs to address and improve DEI in their local environments (Table 2). All programs made substantial efforts to grow the pool of diverse applicants, improve the residency application and interview process, and improve inclusion during residency. All applicants described efforts along the educational continuum, with variability in intensity, reach or breadth, and outcome.

**Table 2.** Themes, Strategies, and Practices to Develop an Organizational Culture That Emphasizes Diversity, Equity, and Inclusion

Target population	Themes, strategies, and practices	Outcomes
Growing the pool of diverse applicants		
Premedical students	Participate in K-12 school-based career, health, or education events (foundational strategy)	Description of K-12 partners (location, percent of students qualifying for free or reduced meals, student demographics)
	Sponsor or participate in outreach and/or partnerships on community college and 4-y college campuses (foundational strategy)	No. and location of events and attendees
	Develop longitudinal bidirectional collaborations (eg, articulation agreements, annual workshops, funded rotations and/or research) with organizations such as historically Black colleges and universities,^17^ minority group–serving institutions,^13^ MESA,^14^ Umoja,^15^ TRIO,^16^ or others working with historically excluded and URiM applicants (aspirational strategy)	No. and location of events and attendees
	Sponsor or participate in outreach events with diversity-focused postbaccalaureate programs (aspirational strategy)	No. of events including postbaccalaureate programs
	Develop formal articulation agreements with diversity-focused postbaccalaureate programs (aspirational strategy)	Articulation agreements and outcomes (if applicable)
Medical students	Staff outreach booth, participate as speakers, and/or provide financial support at regional or national events with large numbers of URiM premedical and medical students (SNMA,^18^ LMSA,^19^ APAMSA,^20^ GLMA^21^) (foundational strategy)	No. of events attended and No. of personnel supported to attend (students, residents, staff, faculty)
	Use social media platforms to celebrate program and/or participate in online networks with other organizations (eg, SNMA,^18^ LMSA,^19^ MiMentor^22^) (aspirational strategy)	Traffic to program website and social media
	Offer funded visting internship and/or subinternship for visiting applicants (foundational strategy)	No. of visiting subinternship participants and percentage of participants who are recruited as interns
	Lead mentorship programs for historically excluded and URiM medical students (aspirational strategy)	Program expectations of faculty and residents and program support for student mentoring activities
Creating a more equitable and inclusive GME recruitment and selection experience		
Applicants	Use mission-driven holistic review for admissions and selection (foundational strategy)	Content and frequency of holistic review training for all admissions committee members; applicant pool diversity, particularly in mission-driven groups; No. and percentage of historically excluded and URiM applicants interviewed, ranked to match, and matched
Interviewers and interviewees	Train interviewers in implicit bias mitigation (foundational strategy)	Percentage of all interviewers trained each year
	Train interviewers on how racism and discrimination impact admission processes and advancement in medical training (foundational strategy)	Percentage of all interviewers trained each year
	Blind interviewers to applicant metrics (aspirational strategy)	Details on which metrics are shared and not shared at which steps in the review of applicants
	Standardize interview questions across interviewers (aspirational strategy)	Interview review forms are coproduced by DEI committee, residents, and faculty
	Include interview questions focused on DEI and health equity (foundational strategy)	DEI and health equity questions are coproduced by DEI committee, residents, and faculty
	Offer resident, faculty, and interviewer dinners during interviews (aspirational strategy)	Historically excluded and URiM applicants who attended diversity dinners
	Include intentional DEI and inclusion strategies during interview day (foundational strategy)	Description of inclusion strategies used
	Intentionally match interviewers to applicants by background, interests, and/or experiences (aspirational strategy)	Percentage of applicants interviewed with matched interviewer
Selected applications	Include historically excluded and URiM faculty, residents, and other stakeholders (nurses, patients, community members) on selection committee (foundational strategy)	DEI composition of selection committee and selection committee charter/mission
	Sponsor second-look events (aspirational strategy)	Percentage of historically excluded and URiM or total attendees who attend second-look event
Improving inclusion during residency		
Residents	Offer a longitudinal curriculum on DEI and health equity for some or all residents (aspirational strategy)	Curriculum details (eg, content, length, required or elective) and participant details
	Offer clinical rotations with medically underserved communities for some or all residents (aspirational strategy)	Rotation details (eg, content, length, required or elective) and participant details
	Support historically excluded and URiM residents through peer groups, trainee DEI conferences, book clubs, grand rounds, and wellness programming (aspirational strategy)	Types of support provided (eg, infrastructure, financial)
	Compensate residents for DEI work above and beyond the residency program requirement (aspirational strategy)	Details of work and compensation source
	Support DEI committee of residents, faculty, staff, and others financially and logistically (foundational strategy)	Composition of committee and how program provides support to committee
	Include DEI committee members on interview committees, ranking committees, and outreach to interviewees (foundational strategy)	Roles and responsibilities of DEI committee across the GME program
	Support resident-led innovations that promote inclusion, such addressing structures and policies surrounding personally experienced and witnessed discrimination and bias (foundational strategy)	Examples and outcomes of resident-led efforts
	Offer historically excluded and URiM trainee leadership opportunities, DEI excellence awards, and/or program or institution-wide DEI day to recognize the unique importance of DEI in their program, institution, or specialty society (aspirational strategy)	Types of trainee leadership opportunities and DEI excellence awards offered
Faculty	Offer explicit retention efforts to improve faculty diversity (foundational strategy)	Types of efforts (research, leadership, mentorship) designed to improve resident retention as faculty and trend of outcomes
	Support faculty to conduct research in and attend professional development offerings related to DEI and health equity (aspirational strategy)	Types of support offered
	Offer faculty mentoring such as affinity groups, mentored research, and joint academic-community recruitments (aspirational strategy)	Programs offered and outcomes
Community	Support community members as key partners in DEI efforts (aspirational strategy)	Community-prioritized programs
	Develop a publicly available mission statement that include DEI (foundational strategy)	Program mission statement

Abbreviations: APAMSA, Asian Pacific American Medical Student Association; DEI, diversity, equity, and inclusion; GLMA, Health Professionals Advancing LGBTQ Equality (previously known as the Gay & Lesbian Medical Association); GME, graduate medical education; K-12, kindergarten through grade 12; LMSA, Latino Medical Student Association; MESA, Mathematics, Engineering, and Science Achievement; SNMA, Student National Medical Association; URiM, underrepresented in medicine.

### Growing the Pool of Diverse Applicants

Graduate medical education programs augmented their outreach efforts to focus on growing the number of historically excluded and URiM students. Residency programs were active in prehealth pathway programs and kindergarten through grade 12 schools with teaching in health classes, participating in career fairs and shadowing, or offering workshops in first aid or suturing.

Practices included outreach to community college students, college and university students, and students enrolled in postbaccalaureate programs. One residency cohosted a day-long premedical conference on a community college campus in partnership with MESA (Mathematics, Engineering, and Science Achievement), a statewide college and career preparation engine that propels student diversity and achievement in science, technology, engineering, and math.^23^

#### Recruiting 

Residents and faculty participated in outreach at events with large numbers of historically excluded and URiM medical (and premedical) students. These included annual or regional meetings of the Student National Medical Association (SNMA),^18^ Latino Medical Student Association (LMSA),^19^ Asian Pacific American Medical Student Association (APAMSA),^20^ and Health Professionals Advancing LGBTQ Equality (previously known as the Gay & Lesbian Medical Association [GLMA]).^21^

One program made annual recruitment trips to historically Black colleges and universities with medical schools.^17^ Mentorship with these students and organizations frequently continued after the meetings. Often residency programs used social media platforms to reach a wide audience.^18,19,22^

#### Visiting Internships and Subinternships

Several programs reported highly successful funded visiting internship and/or subinternship programs for visiting students. Trainees described the funding for travel, housing, and meals as vital to allowing them to choose visiting internships away from their home institution. Funded visiting internships and/or subinternships both increased the programs’ visibility and served as a successful recruitment strategy for the visiting students. Successful visiting programs included clinical immersions, faculty mentorship, and support for students to envision themselves in academic careers. As many as 25% of the visiting students subsequently matched into the residency program.

### Creating a More Equitable and Inclusive GME Recruitment and Selection Experience

#### Innovating the Residency Application Process and Using Holistic Review

Many GME programs cited improvements in their review of applicants as critical to the successful recruitment of historically excluded and URiM residents. Programs described using mission-driven holistic approaches to review applications. For example, one program increased emphasis on the “road traveled,” that is, life experiences and community leadership while de-emphasizing numeric metrics (eg, United States Medical Licensing Examination scores). Another program specifically focused on applicants committed to providing medical care to the diverse populations served by the program by examining applicants’ history of service, leadership, and advocacy.

#### Integrating DEI Into the Residency Interview Process

Once selected for an interview, several strategies were used to improve the interview experience for historically excluded and URiM applicants. In almost all cases, implicit bias training was required for all interviewers. Some programs additionally required interviewer training on how racism and discrimination impact admission processes and advancement in medical training. In some cases, interviewers were blinded to applicant metrics (eg, United States Medical Licensing Examination Step Examination and Medical College Admission Test scores, grade-point average) or intentionally matched to applicants based on shared background, interests, or experiences. At one program, LGBTQIA and pronoun pins were given to applicants as they began the interview day.

To align with the program’s mission statement and to minimize bias in interviews, one program used behavioral interviewing techniques focused on the behaviors the program seeks in residents. Many programs offered an interview day with an intentional DEI and/or inclusion theme. For example, a residency hosted diversity interview days when applicants can opt for a day focused on Black or African American and Latinx applicants. During these expanded interview days, applicants have an opportunity to meet with racial and ethnic minority residents, alumni, and faculty to help them start to build a network of support and encourage them to choose to train there. Other programs used interview questions on DEI and health equity, offered interview day dinners with historically excluded and URiM residents and faculty, and moved away from unstructured interviews to include standard questions that reflect the program’s values, patient population, and clinical practice environment.

Graduate medical education programs with diverse resident cohorts ensured that selection committees were populated with historically excluded and URiM faculty, residents, and other stakeholders such as nurses, patients, and community members. At least 1 program paid stipends to URiM committee members.

Following the interview, some programs reached out to URiM applicants and/or sponsored second-look events. One program created and supported a group of residents and faculty to focus on the recruitment of individuals who define themselves as URM applicants.

### Improving Inclusion During Residency

“Our first month of residency training at ___ was devoted solely to understanding the historic context of social injustice and civil unrest that resulted in the founding of the institution and its hospital.”—from a GME program

#### Curricula, Rotations, and Research

Efforts of GME programs focused on the resident experience and retention of residents as faculty were of equal importance. All programs offered curricula on DEI and health equity for some or all residents. Key features included longitudinal (months to years) curricula and didactics, facilitated dialogues, and skill-building sessions on topics such as microaggressions, systemic racism, and the burden of underrepresentation. Programs frequently engaged residents to lead new initiatives. For example, one residency’s LGBTQIA residency group identified and helped fill a critical and underrecognized gap in the training of residents and fellows by providing formal LGBTQIA health competencies training, creating an inclusive forum for LGBTQIA trainees, formally engaging (other institutional) trainees in an annual Pride event, and contributing to the recruitment of LGBTQIA residents and fellows.

Clinical rotations in medically underserved communities were a common intentional strategy. In some programs, these longitudinal experiences were core to the program mission. For example, 1 program embedded community partnerships and advocacy into the entirety of the clinical experiences. Other programs offered shorter tracks (eg, 1-year or 1-month community immersions) and/or research opportunities on health equity and health disparities. In addition to academic enhancements, programs provided support for historically excluded and URiM residents through peer groups, conferences, book clubs, grand rounds, and wellness programming focused on equity and inclusion.

#### Discrimination and Racism

In recognizing the continuing impact of racism and discrimination on its residents, 1 program created resident-led working groups focused on recruitment and representation, resident education, research, resident well-being, advocacy for underresourced patients and families, and community partnerships. This program has led to system-wide efforts to formalize reporting structures and policies surrounding personally experienced and witnessed discrimination and bias.

#### Addressing the Minority Tax

One program compensated residents for DEI work. Another program made efforts to acknowledge the holy days of the various faiths represented by their trainees, faculty, and staff.

#### Diversity Committees

Most programs had diversity committees composed of residents, faculty, and staff. Some committees were longstanding, but many had been recently developed at the request of trainees. Well-developed diversity committees were actively engaged in outreach, recruitment, and retention efforts. Committee members were included in interview and ranking committees and often offered personalized outreach to interviewees—an effort that was referenced as critical to enhancing inclusion among many of the historically excluded and URiM trainees. Many residency programs and institutions provided financial and logistical support for resident diversity committees.

#### Leadership Opportunities for URiM Trainees and Faculty

Programs offered historically excluded and URiM trainee leadership opportunities, DEI excellence awards, and program or institution-wide DEI days. These efforts recognized the unique importance of DEI in their program, institution, or specialty society.

#### Retaining URiM Residents as Faculty

Programs that reported successful retention of residents as faculty were intentional in their efforts to support trainees through mentorship, inclusion on research projects, and sponsorship for leadership positions (and through creating positions if they did not yet exist). Programs supported faculty to conduct research in and attend professional development offerings related to DEI and health equity. Faculty mentoring included affinity groups such as Minority Men’s Night, mentored research, and joint academic-community recruitments.

### Measuring, Monitoring, and Disseminating Success

A key feature of successful programs was using a data-driven continuous quality improvement approach to measuring, monitoring, and disseminating DEI efforts. Most programs tracked the characteristics of applicants, interviewees, interns, and program graduates to monitor the program’s success in recruiting and retaining for diversity, while others modified their DEI practices only after a single self-review or sentinel event leading to self-review. Strategies included surveys to measure GME graduates’ knowledge, self-efficacy, and self-confidence; to monitor their premedical participants’ transitions into medical school or other health professions careers; and to track GME graduates into practice (ie, monitoring practice type, location, and patient population). Another strategy used publicly available data from the Association of American Medical Colleges’s Electronic Residency Application Service^24^ on residency applicants from US doctor of medicine degree–granting medical schools to ACGME-accredited programs by specialty and self-identified race or ethnicity to compare their percentage of all URiM applicants who applied to their residency program.

Many programs disseminated their successes in meetings and peer-reviewed publications. Many URiM GME program faculty are engaged in national organizations focused on diversity and equity (eg, SNMA, LMSA, APAMSA) or through specialty societies to provide a national focus on recruiting a diverse physician workforce.

## Discussion

Review of applications to the ACGME’s Barbara Ross-Lee, DO, Diversity, Equity, and Inclusion Award identified strategies, themes, and practices for growing the pool of applicants, improving resident recruitment and selection, and enhancing the residency environment. Fifteen strategies were identified as foundational for all programs to consider implementing. Eighteen additional strategies were identified as aspirational targets for programs to consider implementing as part of their robust DEI commitment.

Based on the data reviewed from these exemplars in DEI, GME programs should carefully consider adopting the practices outlined herein. Programs might implement 1 or more foundational strategies targeting each group (eg, from premedical students to applicants and interviewees, to faculty and community) along the continuum. Alternatively, programs with areas of strength might focus on aspirational strategies in their areas of need. Concurrent focus on recruitment, retention, and inclusion is critical. All efforts require institutional commitment to catalyze and sustain program-level practices, particularly in creating resource-intensive continuous quality improvement strategies. US GME programs deserve recognition for innovative efforts to improve DEI in their programs, yet much work remains to be done, particularly in understanding how sponsoring institution policies and practices impede or facilitate GME diversity.

### Limitations

This study has several limitations. First, the applications were from GME programs that self-identified as exemplars in DEI, raising the possibility of selection bias. However, given the limited information in the literature regarding best and emerging strategies used across multiple GME programs, we believe these results have credibility and can be applied to other US GME programs. The number of applications declined from 22 in year 1 to 7 in year 2 for unclear reasons, but in total represent a small but diverse range of specialties, locations, and settings across the US. In addition, there were several areas where promising practices were mentioned but details were lacking, such as reporting discrimination, efforts to retain residents who may experience challenges during training, efforts focused on ethnic populations (eg, Hmong or Vietnamese Cuban), or disabled communities. Furthermore, as these were award applications, there was no mention of lessons learned along the way to becoming exemplars, which merits further study with a potential for broad applicability to the numerous GME programs that may not have considered themselves competitive for this award.

## Conclusions

In this qualitative study of ACGME DEI award applications, we found that GME programs made great efforts to improve DEI. While GME programs attend to their unique strengths and needs, in review of the data and in the opinion of the authors, all GME programs should consider implementing at least 1 foundational effort for each target population, with particular attention to the experience for applicants and interviewees.

## Notes

Supplement.
Data Sharing Statement

## References

[1] Marrast LM, Zallman L, Woolhandler S, Bor DH, McCormick D. Minority physicians’ role in the care of underserved patients: diversifying the physician workforce may be key in addressing health disparities. JAMA Intern Med. 2014;174(2):289-291. doi:10.1001/jamainternmed.2013.12756 24378807

[2] Cooper LA, Roter DL, Johnson RL, Ford DE, Steinwachs DM, Powe NR. Patient-centered communication, ratings of care, and concordance of patient and physician race. Ann Intern Med. 2003;139(11):907-915. doi:10.7326/0003-4819-139-11-200312020-00009 14644893

[3] Keith SN, Bell RM, Swanson AG, Williams AP. Effects of affirmative action in medical schools: a study of the class of 1975. N Engl J Med. 1985;313(24):1519-1525. doi:10.1056/NEJM198512123132406 4069161

[4] Moy E, Bartman BA. Physician race and care of minority and medically indigent patients. JAMA. 1995;273(19):1515-1520. doi:10.1001/jama.1995.03520430051038 7739078

[5] Accreditation Council of Graduate Medical Education. Common program requirements (residency). July 1, 2019. Accessed December 1, 2021. https://www.acgme.org/globalassets/PFAssets/ProgramRequirements/CPRResidency2019.pdf

[6] Accreditation Council for Graduate Medical Education. Barbara Ross-Lee, DO, Diversity, Equity, and Inclusion Award. Accessed March 1, 2022. https://www.acgme.org/what-we-do/initiatives/awards/diversity-and-inclusion-award/

[7] W. G. Kellogg Foundation. Logic model development guide. Updated January 2004. Accessed August 18, 2021. https://www.naccho.org/uploads/downloadable-resources/Programs/Public-Health-Infrastructure/KelloggLogicModelGuide_161122_162808.pdf

[8] Shenton A. Strategies for ensuring trustworthiness in qualitative research projects. Educ Inf. 2004;22(2):63-75. doi:10.3233/EFI-2004-22201

[9] Boyatzis R. Transforming Qualitative Information: Thematic Analysis and Code Development. Sage Publications; 1998.

[10] Dedoose. Version 8.0.35. SocioCultural Research Consultants, LLC. Accessed January 17, 2021. https://dedoose.com/

[11] American Medical Association. FRIEDA database. Accessed December 10, 2022. https://freida.ama-assn.org

[12] Wallace C. 20 Medical schools with the most NIH funding: US News. August 29, 2022. Accessed December 2, 2022. https://www.beckersasc.com/asc-news/20-medical-schools-with-the-most-nih-funding-us-news.html

[13] US Department of Education. Lists of postsecondary institutions enrolling populations with significant percentages of undergraduate minority students. Accessed January 6, 2023. https://www2.ed.gov/about/offices/list/ocr/edlite-minorityinst.html

[14] MESA. Delivering California’s future STEM workforce. Accessed January 6, 2023. https://mesa.ucop.edu

[15] Umoja Community. Umoja promotes student success for all students through a curriculum that is responsive to the legacy of the African and African American Diasporas. Accessed January 6, 2023. https://umojacommunity.org

[16] US Department of Education. Federal TRIO programs. Modified June 9, 2022. Accessed January 6, 2023. https://www2.ed.gov/about/offices/list/ope/trio/index.html

[17] US Department of Education. White House initiative on advancing educational equity, excellence, and economic opportunity through historically Black colleges and universities. Accessed January 6, 2023. https://sites.ed.gov/whhbcu/one-hundred-and-five-historically-black-colleges-and-universities/

[18] Student National Medical Association. Accessed January 6, 2023. https://snma.org

[19] Latino Medical Student Association. Accessed January 6, 2023. https://national.lmsa.net

[20] Asian Pacific American Medical Student Association. Accessed January 6, 2023. https://www.apamsa.org

[21] Health Professionals Advocating LGBTQ Equality. Accessed January 6, 2023. https://www.glma.org

[22] MiMentor. Accessed January 11, 2023. https://www.mimentor.org/

[23] MESA. Delivering California’s future STEM workforce. Accessed April 6, 2022. https://mesa.ucop.edu

[24] Association of American Medical Colleges. AAMC facts: applicants, matriculants, enrollment, graduates, MD-PhD, and residency applicants data. 2021. Accessed December 2, 2022. https://www.aamc.org/data-reports/students-residents/report/facts

