# Supplementary material for: Strategies and Best Practices to Improve Diversity, Equity, and Inclusion Among US Graduate Medical Education Programs
Source: JAMA Netw Open. 2023 Feb 8;6(2):e2255110. doi: 10.1001/jamanetworkopen.2022.55110 (PMC9909494; doi:10.1001/jamanetworkopen.2022.55110)
Supplement: Supplement. — Data Sharing Statement [file jamanetwopen-e2255110-s001.pdf]

Data Sharing Statement

Boatright. Strategies and Best Practices to Improve Diversity, Equity, and Inclusion Among US Graduate Medical Education Programs. *JAMA Netw Open*. Published February 08, 2023. doi:10.1001/jamanetworkopen.2022.55110

Data
Data available: No
